# Retraction Note to: Content validity and psychometric evaluation of functional assessment of chronic illness therapy-fatigue in patients with psoriatic arthritis

**DOI:** 10.1186/s41687-019-0125-2

**Published:** 2019-05-28

**Authors:** David Cella, Hilary Wilson, Huda Shalhoub, Dennis A. Revicki, Joseph C. Cappelleri, Andrew G. Bushmakin, Elizabeth Kudlacz, Ming-Ann Hsu

**Affiliations:** 1 Department of Medical Social Sciences, Northwestern University, 633 N. St. Clair Suite 1900, Chicago, IL 60611 USA; 2 Evidera, Bethesda, MD USA; 3 Evidera, Waltham, MA USA; 4 Pfizer Inc, Groton, CT USA

**Retraction Note to: J Patient Rep Outcomes**

**https://doi.org/10.1186/s41687-019-0094-5**

The publisher has retracted this article [1] because the incorrect version of the article was published in error. The manuscript has been republished as [2]. Springer Nature apologises to readers.

All authors agree to this retraction.
